# Elevated Cardiac Troponin T in Patients with Lupus Myositis Presenting with Noncardiac Chest Pain

**DOI:** 10.1155/2020/8884759

**Published:** 2020-10-23

**Authors:** Guy Katz, Sharon L. Kolasinski, Baskaran Sundaram, Giorgos Loizidis

**Affiliations:** ^1^Division of Rheumatology, Allergy, and Immunology, Department of Medicine, Massachusetts General Hospital, Harvard Medical School, Bulfinch 165, 55 Fruit St., Boston, MA 02114, USA; ^2^Division of Rheumatology, University of Pennsylvania Perelman School of Medicine, Penn Musculoskeletal Center, 3737 Market Street, 8^th^ Floor, Philadelphia, PA 19104, USA; ^3^Department of Radiology, Division of Cardiothoracic Radiology, Thomas Jefferson University, 132 South 10^th^ Street, 10^th^ Floor, The Main Building, Philadelphia, PA 19107, USA; ^4^Division of Rheumatology, Department of Medicine, Thomas Jefferson University, 211 South 9^th^ Street, Suite 600, Philadelphia, PA 19107, USA

## Abstract

Patients with systemic lupus erythematosus (SLE) presenting with chest pain pose a unique diagnostic challenge, with causes ranging from cardiopulmonary disease to esophageal disorders and musculoskeletal chest wall pain. The most common biomarkers for myocardial injury are cardiac troponin T and I (cTnT and cTnI) due to their high sensitivity for the early detection of myocardial infarction. In the idiopathic inflammatory myopathies, cTnT is commonly elevated, and this reflects skeletal muscle breakdown rather than myocardial damage. Similar observations have not been reported in SLE myositis to date. We present two cases of patients with SLE and associated myositis who presented with chest pain and elevated cTnT. Both patients had a normal cTnI, transthoracic echocardiogram, and cardiac magnetic resonance imaging, likely indicating noncardiac chest pain. Clinicians should be aware that the specificity of cTnT might be lower in SLE myositis and that cTnI elevation may be more specific in detecting myocardial insult.

## 1. Introduction

Chest pain is one of the most common complaints seen in emergency departments. In one single-center study, chest pain was the presenting complaint in 15% of visits by patients with systemic lupus erythematosus (SLE) [1].

Patients with SLE presenting with chest pain pose a diagnostic challenge to clinicians. In SLE, the etiology of chest pain can be attributed to pulmonary (pleuritis and pulmonary embolism), esophageal, musculoskeletal (costochondritis, tendonitis, and muscle spasm), and cardiac disease (pericarditis, myocarditis, valvular disease, conduction system abnormalities, microvascular disease, and coronary artery disease).

Systemic lupus erythematosus can result in myocardial injury through accelerated atherosclerosis, direct myocardial inflammation, and microvascular disease [2]. When assessing for myocardial injury in this population, the biomarkers of choice are cardiac troponin I (cTnI) and cardiac troponin T (cTnT), as their high sensitivity can lead to early diagnosis of myocardial infarctions. However, in the idiopathic inflammatory myopathies (IIMs), the specificity of cTnT for myocardial injury has been reported to be reduced. Indeed, concomitant elevations in creatine kinase (CK) and cTnT have been described in patients with IIMs without cardiac involvement [3]. While not a part of formal classification criteria, many patients with SLE experience myositis as part of their disease process [4]. Nevertheless, the effect of myositis on the utility of cardiac biomarkers in patients with SLE has not been established.

We report the first two cases, to our knowledge, of patients with SLE and associated muscle enzyme elevations who presented with chest pain and elevated cTnT, but in whom further diagnostic evaluation with transthoracic echocardiogram (TTE), electrocardiogram (ECG), and cardiac magnetic resonance imaging (MRI) showed no evidence of myocardial injury, ischemia, or inflammation.

## 2. Case No. 1

A 21-year-old woman with a past medical history of SLE with associated myositis and myocarditis presented to the emergency department in 2018 with recurrent pleuritic chest pain.

The patient was first diagnosed with SLE in 2016. Her presenting symptoms were polyarthritis, proximal muscle weakness, alopecia, and chest pain. Initial serologies revealed an antinuclear antibody (ANA) at a titer of 1 : 20,480 with positive anti-Smith, anti-double-stranded deoxyribonucleic acid (dsDNA), antiribonucleoprotein, anti-Ro, and anti-La antibodies as well as a low C4 complement and low-normal C3 complement. Other tests for myositis-specific antibodies were negative (Table 1). Computed tomography of the chest was negative for pulmonary embolism or parenchymal or pleural lung disease.

**Figure 1:**
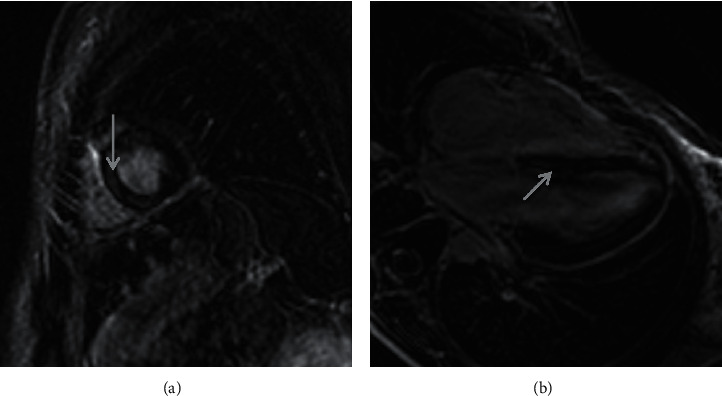
Initial T1-weighted delayed gadolinium-enhanced magnetic resonance images in (a) short axis and (b) four-chamber views demonstrating patchy midmyocardial delayed contrast enhancement in the anterior, inferior, and septal walls (arrows).

Cardiac evaluation during her initial presentation revealed an elevated cTnT, CK, and creatine kinase-muscle/brain (CK-MB). cTnI was not checked during this admission. Electrocardiogram showed sinus tachycardia, and TTE did not reveal any wall motion abnormalities or valvular disease. Out of concern for myocardial involvement of SLE, cardiac MRI was obtained and revealed small patchy areas of midmyocardial delayed gadolinium enhancement in the anterior and inferior walls as well as the septum—findings that were consistent with myocarditis (Figure 1). The patient was given 1,000 mg of intravenous methylprednisolone daily for three days and then discharged home on hydroxychloroquine, mycophenolate mofetil, and 1 mg/kg daily of prednisone with a slow taper. Her chest pain and articular symptoms quickly resolved.

In 2018, two years after her initial diagnosis, the patient presented with sharp substernal pleuritic chest pain radiating to the back. The pain was not reproducible on palpation. Accompanying symptoms at that time included polyarthritis, Raynaud's phenomenon, hair loss, and proximal muscle weakness. Laboratory workup to assess lupus disease activity showed an elevated erythrocyte sedimentation rate (83 mm/hr, reference range 0–20) and anti-dsDNA antibody (544 U/mL, reference range 0–200) as well as low C3 and C4 complement levels (79 mg/dL (reference range: 88–201) and 3 mg/dL (reference range: 10–44), respectively).

**Figure 2:**
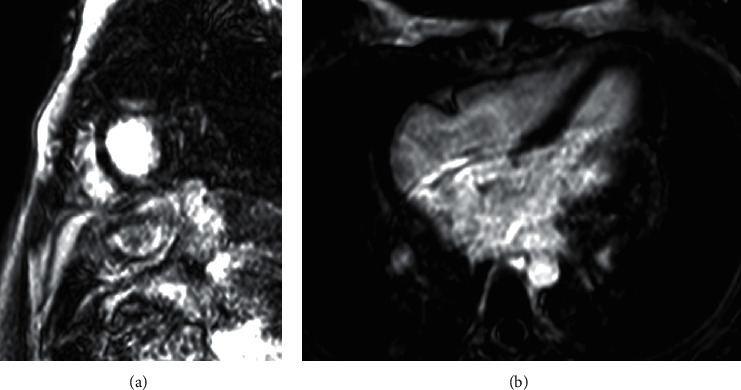
Repeat T1-weighted delayed gadolinium-enhanced magnetic resonance images in (a) short axis and (b) four-chamber views demonstrating the resolution of previously seen delayed contrast enhancement.

Cardiac evaluation during this presentation revealed a high-sensitivity cTnT of 31 ng/L (reference range: <19) and a CK of 320 IU/L (reference range: 25–185), whereas cTnI was undetectable (reference range: <0.05 ng/mL). Her ECG showed sinus tachycardia, and TTE was again normal. Given the prior history of myocarditis, a second cardiac MRI was obtained, this time showing no evidence of myocardial inflammation (Figure 2). The patient's dose of prednisone was briefly increased to 1 mg/kg daily, and her polyarthralgia and chest pain resolved. Her chest pain this time was considered to represent costochondritis.

## 3. Case No. 2

A 24-year-old female with a past medical history of SLE presented in 2018 to the emergency department with episodic substernal chest pain.

She initially presented in 2016 with polyarthritis, a malar rash, and Raynaud's phenomenon. Laboratory studies revealed a positive ANA at a 1 : 320 titer with a speckled pattern and elevated Smith and ribonucleoprotein antibodies. The patient was started on hydroxychloroquine.

In 2018, she developed episodic, substernal, nonexertional, pleuritic chest pain. Computed tomography angiography of the chest showed no evidence of pulmonary embolism. Cardiac workup revealed a cTnT of 0.158 ng/mL (reference range: <0.03), which increased to 0.214 over the next day, and a CK-MB of 58.1 ng/mL (reference range: 0–5); however, cTnI was undetectable (reference range: 0–0.08 ng/mL). Aldolase and CK were elevated as well (13 U/L (reference range: 1–8) and 1157 U/L (reference range: 38–234), respectively), though the patient had no appreciable muscle weakness. Electrocardiogram was unremarkable, and TTE revealed no wall motion abnormalities. Cardiac MRI showed no delayed gadolinium enhancement to indicate myocarditis. Serologic testing revealed a weakly positive Ro-52 antibody of 46 units (reference range: 0–40), but tests for myositis-specific antibodies were negative (Table 1). She was treated with prednisone at a dose of 30 mg daily, and her symptoms resolved. Her chest pain was attributed to possible costochondritis or esophageal spasm.

## 4. Discussion

We report two patients with SLE with chest pain and elevated CK and cTnT but normal cTnI levels. A further extensive workup for myocardial disease including ECG, TTE, and cardiac MRI was unremarkable. We sought to illustrate that elevation in cTnT in a patient with SLE and coexisting myositis may be secondary to skeletal inflammatory muscle disease rather than myocardial inflammation if the cTnI is normal. In turn, lupus-related myositis may follow the paradigm of the IIMs, in which cTnI is rarely elevated without cardiac involvement, and an abnormal elevation in cTnI should raise suspicion for myocardial injury [3].

Myocardial involvement in SLE requires clinical attention, as it can progress to cardiac arrhythmias, congestive heart failure, and cardiogenic shock [5–7]. Cardiac troponin T and troponin I are the biomarkers of choice for the detection of myocardial injury, as they are the most sensitive and cardiac-specific laboratory measures of myocardial injury currently available.

In the IIMs, cTnT has been shown to be elevated with or without CK, even in the absence of cardiac involvement [3, 8]. This is thought to be because cTnT, and not cTnI, is expressed by skeletal muscle, so it is not entirely specific to cardiac myocytes [9, 10]. Furthermore, elevated cTnI in patients with IIMs has had the highest positive predictive value for the detection of myocardial inflammation, as elevations in cTnI appear not to correlate with skeletal muscle inflammation [8, 11]. Similar findings have been observed in skeletal myopathies, in which cTnT was commonly elevated, but elevations in cTnI were rare [12]. This is thought to be due to a combination of cross-reactivity of the cTnT assay with skeletal troponin T and increased expression of cTnT by skeletal muscle under chronic stress [12, 13].

When evaluating a patient with SLE with elevated CK, expansion of immunologic testing with myositis- and scleroderma-related antibodies should be considered, as it may be a sign of an overlap syndrome. When myocardial involvement is suspected, careful examination of all patient data by clinicians is required, from clinical history to subtle ECG changes and TTE findings. Measuring cTnT and cTnI provides complementary information. Elevations in highly sensitive cTnT assays have been correlated with subclinical diffuse myocardial fibrosis and inflammation identified by MRI [14]. Additionally, cTnT elevations can be seen in active myositis without detectable myocardial inflammation, as presented in this report. Elevation of cTnI should prompt further diagnostic workup, as this is less likely to be observed in the absence of myocardial injury [3]. Cardiac MRI has also become an important diagnostic modality in detecting myocardial disease in lupus [14–17]. Even in the absence of TTE abnormalities, cardiac MRI can demonstrate lesions, including myocarditis and vasculitis as well as hemodynamic parameters such as ventricular stiffness [14–16].

This is the first report to our knowledge that aims to illustrate that elevations in cTnT in patients with lupus myositis did not reflect myocardial injury—as both patients had a normal ECG, TTE, and cardiac MRI—and was instead due to active skeletal muscle disease. Additionally, while cTnT was elevated, cTnI was normal in both cases. Further research is needed to investigate the specificity and sensitivity of cTnT and cTnI in detecting silent or symptomatic myocardial injury in the setting of inflammatory muscle disease in SLE.

## Figures and Tables

**Table 1:** Autoimmune antibody profiles for patients 1 and 2.

Antibody	Patient 1		Patient 2	
	Value	Reference	Value	Reference
ANA	>1 : 20.480	Negative	1 : 320	Negative
ANA pattern	Speckled	N/A	Speckled	
Anti-dsDNA	36	0–29	1	0–9
Scl 70	0.2	0–0.9		
SSA	0.4	0–0.9	0.3	0–0.9
SSB	0.1	0–0.9	<0.2	0–0.9
Smith/RNP	Positive	Negative	>8.0	0–0.9
Anticardiolipin IgM	7	0–12.0		
Anticardiolipin IgG	3.5	0–11.0	3.0	0–11.0
Beta-2 glycoprotein IgM	3	0–12.9	0.5	0–12.9
Beta-2 glycoprotein IgG	0.5	0–7.9	0.5	0–7.9
DRVVT screen	Negative	Negative	Negative	Negative
PL-7	Negative	Negative	Negative	Negative
PL-12	Negative	Negative	Negative	Negative
Mi-2	Negative	Negative	Negative	Negative
Ku	Negative	Negative	Negative	Negative
EJ	Negative	Negative	Negative	Negative
OJ	Negative	Negative	Negative	Negative
SRP	Negative	Negative	Negative	Negative
Jo-1	Negative	Negative	Negative	Negative
SSA-52			46	0–40
U1 RNP			54	0–40
SSA-60			Negative	Negative
PM			Negative	Negative
U2 snRNP			Negative	Negative
P155/140			Negative	Negative
TIF-1 gamma			Negative	Negative
SAE-1			Negative	Negative
MDA-5			Negative	Negative
NXP-2			Negative	Negative

## References

[1] Modi M., Ishimori M. L., Sandhu V. K., Wallace D. J., Weisman M. H. (2015). Chest pain in lupus patients: the emergency department experience. *Clinical Rheumatology*.

[2] Miner J. J., Kim A. H. J. (2014). Cardiac manifestations of systemic lupus erythematosus. *Rheumatic Disease Clinics of North America*.

[3] Aggarwal R., Lebiedz-Odrobina D., Sinha A., Manadan A., Case J. P. (2009). Serum cardiac troponin T, but not troponin I, is elevated in idiopathic inflammatory myopathies: figure 1. *The Journal of Rheumatology*.

[4] Liang Y., Leng R.-X., Pan H.-F., Ye D.-Q. (2017). Associated variables of myositis in systemic lupus erythematosus: a cross-sectional study. *Medical Science Monitor*.

[5] Rebelato J. B., Silveira C. F. D. S. M. P. D., Valadão T. F. C., Reis R. M., Bazan R., Bazan S. G. Z. (2018). Myocarditis with cardiogenic shock as the first manifestation of systemic lupus erythematosus. *Arquivos Brasileiros de Cardiologia*.

[6] Thomas G., Cohen Aubart F., Chiche L. (2017). Lupus myocarditis: initial presentation and longterm outcomes in a multicentric series of 29 patients. *The Journal of Rheumatology*.

[7] Jia E., Geng H., Liu Q. (2018). Cardiac manifestations of Han Chinese patients with systemic lupus erythematosus: a retrospective study. *Irish Journal of Medical Science*.

[8] Lilleker J. B., Diederichsen A. C. P., Jacobsen S. (2018). Using serum troponins to screen for cardiac involvement and assess disease activity in the idiopathic inflammatory myopathies. *Rheumatology*.

[9] Bodor G. S., Porterfield D., Voss E. M., Smith S., Apple F. S. (1995). Cardiac troponin-I is not expressed in fetal and healthy or diseased adult human skeletal muscle tissue. *Clinical Chemistry*.

[10] Bodor G. S., Survant L., Voss E. M., Smith S., Porterfield D., Apple F. S. (1997). Cardiac troponin T composition in normal and regenerating human skeletal muscle. *Clinical Chemistry*.

[11] Kiely P. D. W., Bruckner F. E., Nisbet J. A. (2000). Serum skeletal troponin I in inflammatory muscle disease: relation to creatine kinase, CKMB and cardiac troponin I. *Annals of the Rheumatic Diseases*.

[12] Schmid J., Liesinger L., Birner-Gruenberger R. (2018). Elevated cardiac troponin T in patients with skeletal myopathies. *Journal of the American College of Cardiology*.

[13] Vroemen W. H. M., De Boer D., Streng A. S., Mingels A. M. A., Meex S. J. R. (2018). Elevated cardiac troponin T in skeletal myopathies. *Journal of the American College of Cardiology*.

[14] Winau L., Hinojar Baydes R., Braner A. (2018). High-sensitive troponin is associated with subclinical imaging biosignature of inflammatory cardiovascular involvement in systemic lupus erythematosus. *Annals of the Rheumatic Diseases*.

[15] Mavrogeni S., Koutsogeorgopoulou L., Markousis-Mavrogenis G. (2018). Cardiovascular magnetic resonance detects silent heart disease missed by echocardiography in systemic lupus erythematosus. *Lupus*.

[16] Burkard T., Trendelenburg M., Daikeler T. (2018). The heart in systemic lupus erythematosus—a comprehensive approach by cardiovascular magnetic resonance tomography. *PLoS One*.

[17] Mavrogeni S., Bratis K., Markussis V. (2013). The diagnostic role of cardiac magnetic resonance imaging in detecting myocardial inflammation in systemic lupus erythematosus. Differentiation from viral myocarditis. *Lupus*.

